# Impact of the first-pass pulmonary vein isolation on ablation outcomes in persistent atrial fibrillation

**DOI:** 10.3389/fcvm.2025.1588716

**Published:** 2025-06-05

**Authors:** Chenxu Luo, Bing Leng, Xinzhi Yu, Xianfeng Du, Huimin Chu, Shenyuan Zhou, Caijie Shen, Mingjun Feng, Yongxing Jiang, He Jin, Guohua Fu, Lipu Yu, Binhao Wang, Yibo Yu, Weidong Zhuo, Fang Gao, Yin Xu, Yijun Sun, Jiating Dai, Luigi Di Biase

**Affiliations:** ^1^ Arrhythmia Center, The First Affiliated Hospital of Ningbo University, Ningbo First Hospital, Ningbo, China; ^2^ Key Laboratory of Precision Medicine for Atherosclerotic Diseases of Zhejiang Province, The First Affiliated Hospital of Ningbo University, Ningbo, China; ^3^ Health Science Center, Ningbo University, Ningbo, China; ^4^ Department of Cardiology, Ningbo Taikang Hospital, Ningbo, China; ^5^ Montefiore Medical Center, Albert Einstein College of Medicine, New York, NY, United States

**Keywords:** persistent atrial fibrillation, pulmonary vein isolation, first-pass isolation, radiofrequency ablation, ablation outcome

## Abstract

**Background:**

The achievement of first-pass isolation (FPI) during pulmonary vein isolation (PVI) generally serves as a reliable marker of lesion quality in initial radiofrequency encirclement and predicts favorable procedural outcomes. This study sought to evaluate the impact of the FPI on the long-term clinical outcomes in persistent atrial fibrillation (PeAF) patients undergoing radiofrequency ablation.

**Methods:**

We conducted a retrospective analysis of 346 patients with PeAF who were divided into three groups: patients with FPI in bilateral PVs (BOTH group, *n* = 197), those with FPI in either ipsilateral PVs (EITHER group, *n* = 92), and those without FPI in bilateral PVs (NEITHER group, *n* = 57). Achieving FPI in at least one of the two ipsilateral PVs (at least ipsilateral FPI, IFPI) was utilized as a metric for evaluation. The primary endpoint was freedom from atrial tachyarrhythmias (ATAs) lasting longer than 30s beyond the blanking period. Baseline characteristics, procedural results and long-term clinical outcomes were compared among the groups.

**Result:**

The FPI was effectively achieved in 251 left PVs (72.5%) and 235 right PVs (67.9%). After a median follow-up of 658(402, 970) days, the NEITHER group exhibited less freedom from ATAs recurrence than the BOTH group (57.9% vs. 75.1%, *P* < 0.001) or the EITHER group (57.9% vs. 70.7%, *P* = 0.036). IFPI was an independent predictor of freedom from ATAs recurrence in PeAF patients undergoing their initial ablation (HR, 0.46; 95% CI, 0.29–0.74; *P* = 0.001).

**Conclusion:**

Achieving FPI for PVI remained a significant association with improved ablation outcomes in PeAF patients, wherein IFPI served as an important determinant.

## 1 Introduction

Pulmonary vein isolation (PVI) serves as an efficacious treatment for paroxysmal atrial fibrillation (PAF), especially in symptomatic patients resistant to medical treatment (1). Whilst most triggers located in the pulmonary veins (PVs) drive PAF, persistent forms are associated with variable interaction between triggers and substrate comprised of atrial and PV electrical and structural remodeling (2). Catheter ablation for persistent atrial fibrillation (PeAF) is more challenging and is associated with less favorable outcomes. Despite guidelines and expert consensus advocating for extensive ablation beyond PVI in managing PeAF (1), multiple randomized controlled trials indicated no reduction in the rate of recurrent AF when adjunctive ablation strategies like linear ablation or complex fractionated atrial electrograms (CFAE) ablation were performed in addition to PVI (3–7). However, the reason why patients with PeAF do not benefit from extra-PV ablation remains somewhat ambiguous.

First-pass isolation (FPI) for PVI signals a high-quality lesion set primarily produced in the initial radiofrequency encirclement, hence minimizing the need for touch-up applications. Its achievement is highly indicative of favorable long-term clinical outcomes, explicitly demonstrated within the PAF population (8, 9). The complex mechanisms of initiation and maintenance in PeAF, coupled with various substrate modification strategies in addition to PVI, culminate in an unclear impact of FPI on the success rate of PeAF ablation. This necessitates a deeper comprehension of the efficacy of durable PVI in PeAF management, especially in “early-stage” PeAF. Consequently, our study strives to evaluate the impact of the FPI for PVI on the long-term clinical outcomes in PeAF patients undergoing catheter ablation.

## 2 Methods

### 2.1 Study population

This was a retrospective cohort study. Consecutive patients undergoing radiofrequency ablation (RFA) for drug-refractory PeAF (continuous AF episode lasting longer than 7 days but less than 1 year) at the Arrhythmia Center of the First Affiliated Hospital of Ningbo University between September 2020 and September 2023 were included for analysis.

**Figure 1:**
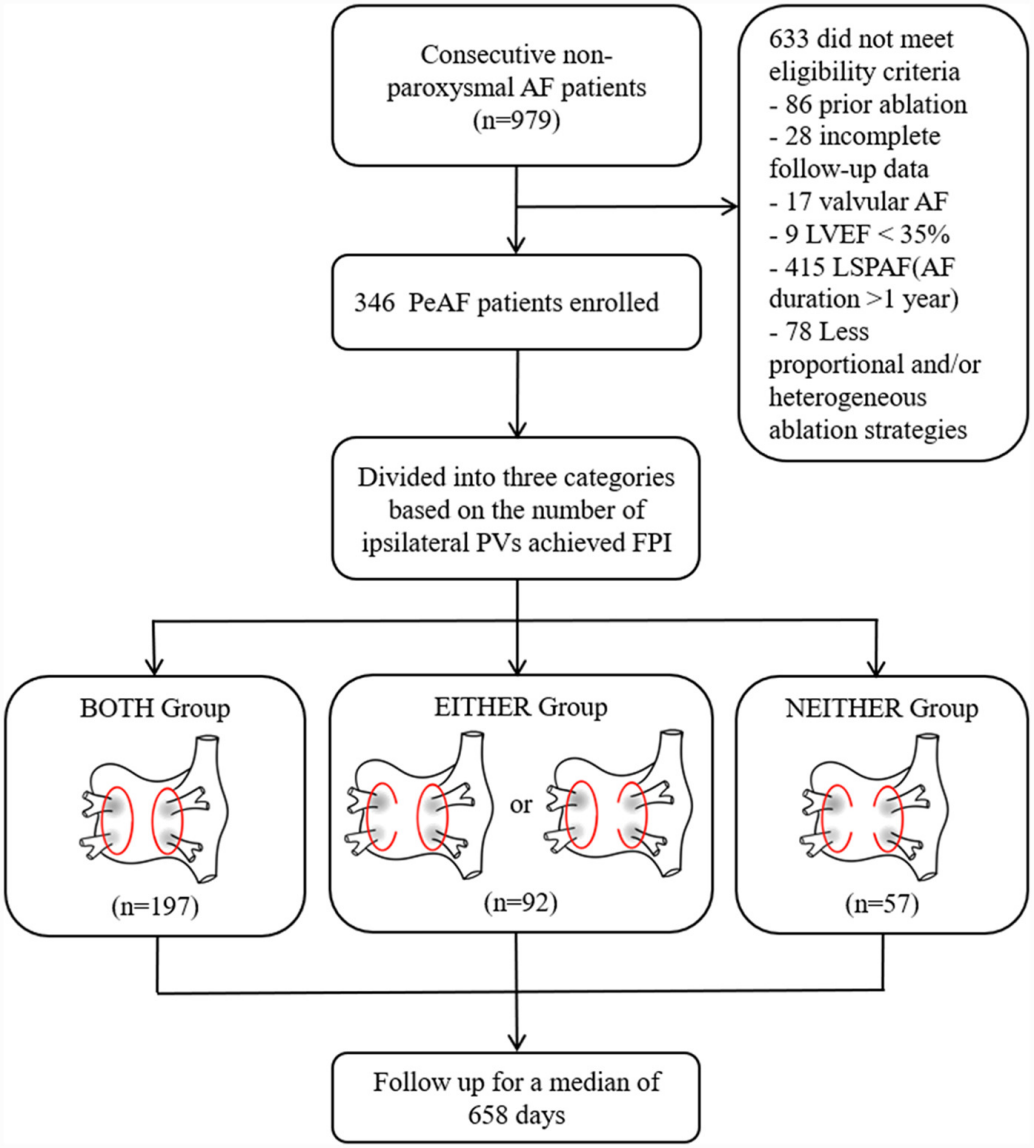
Study flowchart. Abbreviations: AF, atrial fibrillation; PeAF, persistent atrial fibrillation; LSPAF, long-standing persistent atrial fibrillation; LVEF, left ventricular injection fraction; PV, pulmonary vein; PVI, pulmonary vein isolation; FPI, first-pass isolation; BOTH group, patients with FPI in bilateral PVs group; EITHER group, patients with FPI in either ipsilateral PVs group; NEITHER group, patients without FPI in bilateral PVs group.

The study population was divided into three categories based on the number of ipsilateral PVs achieved FPI: patients with FPI in bilateral PVs (BOTH group), those with FPI in either ipsilateral PVs (EITHER group), and those without FPI in both ipsilateral PVs (NEITHER group). The exclusion criteria were as follows: (1) age <18 years old; (2) left ventricular ejection fraction <35%; (3) valvular AF, which refers to patients with severe mitral stenosis, artificial heart valves or valve repair; (4) prior left-sided catheter or surgical ablation procedures; (5) incomplete follow-up data; (6) long-standing persistent atrial fibrillation (LSPAF, continuous AF episode lasting longer than 1 year, in whom rhythm control management is being pursued) and (7) Less proportional and/or heterogeneous extra-PV ablation strategies. The study process and patient enrollment are depicted in Figure 1.

All patients provided written informed consent for AF ablation. In line with the Declaration of Helsinki's ethical principles, this study received approval from the hospital's Ethics Committee, with informed consent waived for the observational study due to anonymized data.

### 2.2 Radiofrequency ablation and first-pass pulmonary vein isolation

A transesophageal echocardiogram (TEE) was performed within 48 h before the procedure to confirm the absence of a thrombus in the left atrium (LA) and left atrial appendage. Antiarrhythmic drugs (AADs), except Amiodarone, were discontinued for five half-lives before the procedure. RFA was performed under deep conscious sedation and local anesthesia. After femoral venous access, intravenous heparin was administered to maintain an intraprocedural activated coagulation time (ACT) of 250–350 s. A steerable deca-polar catheter was introduced into the coronary sinus vein for intracardiac recording. Intracardiac electrograms were recorded using the multi-channel electrophysiological system (EP-Workmate, Abbott, USA). Three-dimensional electroanatomic mapping (EAM) systems (CARTO 3 version 6, Biosense Webster, USA) provided the operators with anatomic reconstructions. Multipolar mapping catheters (Pentaray Nav, Biosense-Webster, USA) were used for geometry reconstruction and mapping after respiratory gating settings. An open irrigated-tip contact force (CF)-sensing catheter (Thermocool SmartTouch, Biosense-Webster, USA) was employed to deliver RFA. Ablation lesions were delivered at quantitative indices [for LA anterior/roof segments: ablation index (AI) of 500–550; for LA posterior/inferior segments: AI of 350–400] in conjunction with an interlesion distance not more than 5 mm and a target CF of 5–20 g, following the CLOSE protocol (10).

All patients underwent PVI. Contiguous, encircling, ablation lesions were created around ipsilateral PV pairs to achieve isolation. Acute PVI was defined as a bidirectional conduction block between the PVs and LA following sequentially applying point-by-point ablation at the PV antrum. An entrance block was identified by the absence of PV potentials recorded by the multipolar mapping catheter. Electric cardioversion was applied to restore the sinus rhythm if the AF could not be terminated spontaneously. Intra-PV pacing (output of 20 mA at a pulse width of 2 ms) without capture of the LA was performed to identify the exit block after the restoration of sinus rhythm. In cases where isolation was not achieved, conduction gaps were tagged on the EAM system, and additional RF applications with the same AI targets were delivered at the gap sites. After a waiting period of 20 min since the last ablation, all PVs were assessed again and adenosine testing was performed to reveal dormant electrical conduction. FPI was defined as the completion of first-pass circumferential ablation lesion sets and isolation of ipsilateral PV without the need for additional touch-up ablations at the intervenous carina or gaps in the initial circumferential ablation circle. Given that the previous study has mentioned that FPI achievements in at least one of the two ipsilateral PVs (at least ipsilateral FPI, IFPI, inclusive of FPI in bilateral PVs and either ipsilateral PVs) to be significantly associated with ablation outcomes in the PAF population (9), its integration into our research was deemed necessary. PVI alone, PVI Plus LA roof line (LARL), and PVI Plus posterior wall isolation (PWI) were included as primary ablation strategies. The study was conducted by four experienced practitioners at our center with extensive experience performing AF ablation procedures, performing more than 200 procedures individually per year.

### 2.3 Post-ablation management and follow-up

Oral anticoagulants (OAC) and AAD were prescribed within the 90-day blanking period after ablation. Continuous OAC was recommended following the current guideline if the patient was at high risk of thromboembolism. AAD was discontinued after the blanking period. Outpatient clinic visits were scheduled at 1, 3, and 6 months after the procedure, followed by biannual visits. Each visit included physical examinations, 12-lead ECG, and 24-h Holter monitoring. Patients reporting symptoms of palpitations underwent a 24-h Holter recording and were evaluated for the possibility of arrhythmia recurrence. The primary study endpoint was the recurrence of atrial tachyarrhythmias (ATAs), defined as documented AF and organized atrial tachycardias (AT, including atrial flutter and tachycardia) lasting ≥30 s beyond the 90-day blanking period.

### 2.4 Statistical analysis

Variables conforming to a normal distribution were reported as mean ± standard deviation (SD), while non-normally distributed variables were reported as median and interquartile range (IQR). Dichotomous and categorical variables were presented in percentages. The Kruskal-Wallis test or ANOVA was used for continuous variable comparisons, and the chi-square test or Fisher's exact test for categorical variable comparisons. Freedom from ATAs/AF/AT recurrence was analyzed using the Kaplan–Meier method. Univariate and multivariate Cox proportional hazard models were used to evaluate predictors of ATAs/AF/AT recurrences. Subgroup analysis of the primary endpoint indicating hazard ratios and *P*-values for interaction was performed based on Cox regression analysis. Subgroup selection was determined by clinical experience and potential risk factors suggested by Cox regression. Restricted cubic spline was performed on continuous variables to find the appropriate thresholds. Propensity score matching was employed to address potential confounding. Propensity scores were calculated using logistic regression, incorporating variables such as age, gender, BMI, duration of AF persistence and left atrium diameter (LAD). Matched cohorts were created using nearest neighbor matching with a caliper of 0.2 standard deviations of the logit of the propensity score. IBM SPSS Statistics (version 26; SPSS Inc, USA) was employed for statistical analyses, and figures were created with GraphPad Prism (version 9.1; GraphPad Software, USA).

## 3 Results

### 3.1 Baseline characteristics

A total of 346 patients were available for analysis, which included 197 patients in the BOTH group, 92 in the EITHER group, and 57 in the NEITHER group. A detailed presentation of the demographic and baseline characteristics is available in Table 1. Patients with PeAF had a median AF episode duration of 3.0 (1.0, 6.0) months with 162 (46.8%) patients experiencing duration for less than 3 months. No significant differences were observed among the three groups for the all variables.

**Table 1:** Demographics and baseline characteristics.

Variables	BOTH (*n* = 197)	EITHER (*n* = 92)	NEITHER (*n* = 57)	*P* value
Demographics				
Sex, male, *n* (%)	130 (66.0)	58 (61.7)	44 (77.2)	0.181
Age, mean ± SD (years)	65.4 ± 9.5	63.7 ± 8.9	62.8 ± 9.9	0.107
BMI, mean ± SD (kg/m^2^)	24.4 ± 3.5	24.4 ± 3.0	25.2 ± 3.8	0.323
CV score, median (IQR)	3 (1, 4)	2 (1, 4)	2 (1, 4)	0.443
HB score, median (IQR)	1 (1, 2)	1 (1, 2)	1 (1, 2)	0.479
Duration of AF persistence, median (IQR) (months)	3.0 (1.0, 6.0)	2.5 (1.0, 7.8)	3.0 (1.0, 7.5)	0.179
Echocardiographic index				
LAD, mean ± SD (mm)	42.5 ± 5.1	42.0 ± 5.7	42.6 ± 4.3	0.666
LVEF, mean ± SD (%)	61.1 ± 8.0	61.4 ± 8.0	59.8 ± 9.0	0.482
Comorbidities, *n* (%)				
Hypertension	110 (55.8)	50 (54.3)	31 (54.4)	0.963
Diabetes mellitus	24 (12.2)	16 (17.4)	10 (17.5)	0.386
History of stroke/TIA	22 (11.2)	16 (17.4)	7 (12.3)	0.336
Chronic heart failure	53 (26.9)	15 (16.3)	10(17.5)	0.082

Values are given as *n* (%), mean ± SD, or median (first quartile and third quartile) unless otherwise indicated.

Abbreviations: BMI, body mass index; CV, CHA2DS2-VASc; HB, HAS-BLED; AF, atrial fibrillation; LAD, left atrium diameter; LVEF, left ventricular ejection fraction; TIA, transient ischemic attack.

### 3.2 The first-pass pulmonary vein isolation and touch-up sites

The PVI was successfully performed in all patients. Residual conduction gaps present upon completion of the encircling lesion sets were ablated, resulting in bilateral PVI. The FPI was effectively achieved in 251 left PVs (72.5%) and 235 right PVs (67.9%) in patients with PeAF. The spatial distribution of the sites for touch-up RF application in the case of non-FPI is illustrated in Supplementary Figure S1. Additional RF lesions to eliminate conduction gaps were mostly located at the carina between ipsilateral PVs. No significant difference in the proportion of different ablation strategies across the three groups (*P* > 0.05; Table 2).

**Table 2:** Ablation strategies and first-pass pulmonary vein isolation rates.

Variables	Total (*n* = 346)	BOTH (*n* = 197)	EITHER (*n* = 92)	NEITHER (*n* = 57)
PVI alone	103	57 (55.3)	28 (27.2)	18 (17.5)
PVI + LARL	127	71 (55.9)	37 (29.1)	19 (15.0)
PVI + PWI	116	69 (59.5)	27 (23.3)	20 (17.2)
*P* value	–	0.791	0.579	0.845

Values are given as *n* (%).

Abbreviations: PVI, pulmonary vein isolation; LARL, left atrial roof line; PWI, posterior wall isolation.

### 3.3 Follow-up and redo ablation findings

**Figure 2:**
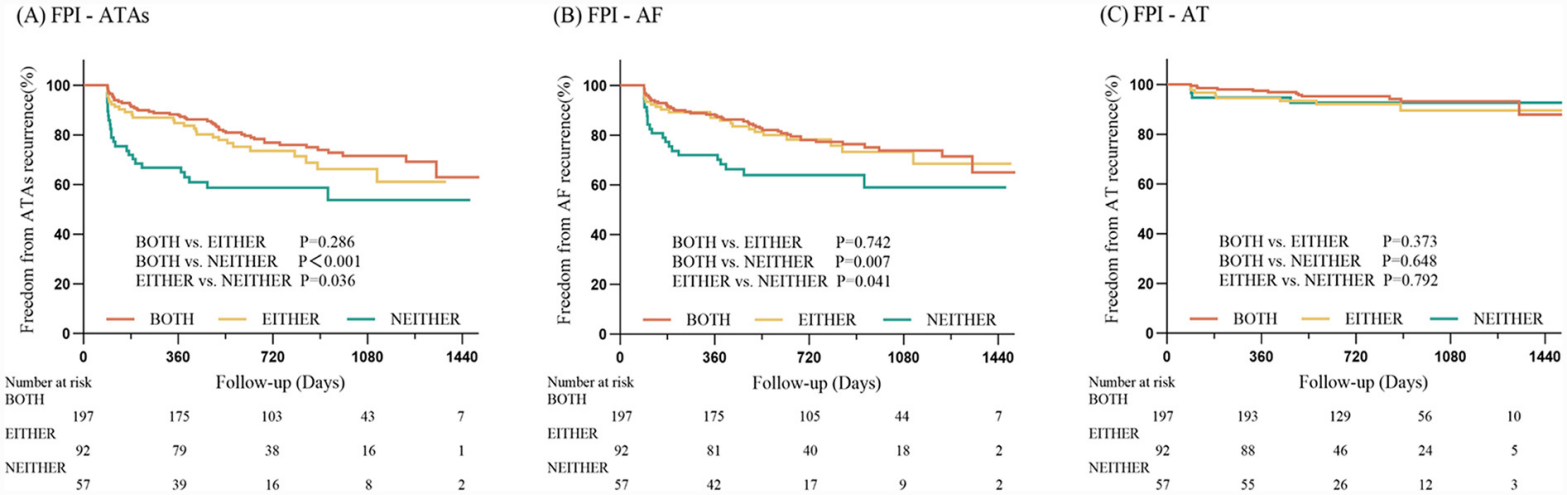
Kaplan–Meier analysis. Comparison of freedom from ATAs **(A)**, AF **(B)** or AT **(C)** recurrences among PeAF patients with FPI achieved in different numbers of ipsilateral PVs. Abbreviations: PeAF, persistent atrial fibrillation; ATAs, atrial tachyarrhythmias; AF, atrial fibrillation; AT, atrial tachycardia.

The median duration of follow-up after the ablation procedure was 658 (402, 970) days. As depicted in Figures 2A,B, the Kaplan–Meier survival analysis indicated that the NEITHER group exhibited less freedom from ATAs and AF recurrence compared to the BOTH group (ATA: 57.9% vs. 75.1%, log-rank *P < * 0.001; AF: 63.2% vs. 76.6%, log-rank *P* = 0.007) or the EITHER group (ATAs: 57.9% vs. 70.7%, log-rank *P* = 0.036; AF: 63.2% vs. 76.1%, log-rank *P* = 0.041). However, there was no statistically significant difference in AT-free survival among the groups (BOTH vs. EITHER vs. NEITHER, 93.9% vs. 91.3% vs. 93.0%; all log-rank *P* > 0.05; Figure 2C). After matching, the baseline characteristics between PVI alone and PVI Plus LARL, as well as PVI alone and PVI Plus PWI cohorts, were well balanced. It demonstrated a similar pattern in freedom from ATAs recurrence between PVI alone and PVI Plus LARL (69.5% vs. 72.6%, log-rank *P* = 0.575; Supplementary Figure S2A). Similarly, there was no significant difference between PVI alone and PVI Plus PWI (67.9% vs. 69.1%, log-rank *P* = 0.892; Supplementary Figure S2B).

Univariate Cox regression analysis revealed that LAD (HR, 1.06; 95% CI, 1.02–1.10; *P* = 0.004), achieving FPI in the left PVs (HR, 0.59; 95% CI, 0.39–0.89; *P* = 0.011), achieving FPI in the right PVs (HR, 0.58; 95% CI, 0.39–0.87; *P* = 0.008), BOTH (HR, 0.62; 95% CI, 0.42–0.92; *P* = 0.016), and IFPI (HR, 0.47; 95% CI, 0.29–0.74; *P* = 0.001) were significantly associated with ATAs recurrence in PeAF patients undergoing their initial RFA. In multivariate Cox regression analysis, LAD (HR, 1.05; 95% CI, 1.01–1.10; *P* = 0.016) and IFPI (HR, 0.46; 95% CI, 0.29–0.74; *P* = 0.001) were independent predictors of ATAs recurrence after adjustment for several factors including gender, age, BMI, duration of AF persistence and ablation strategies (Table 3).

**Table 3:** Univariate and multivariate Cox regression analyses for atrial tachyarrhythmias recurrence.

Baseline variable	Univariate analysis		Multivariate analysis	
	HR (95%CI)	*P* value	HR (95% CI)	*P* value
Male	1.06 (0.70–1.62)	0.771	1.02 (0.66–1.57)	0.943
Age	1.01 (0.99–1.03)	0.367	1.01 (0.99–1.03)	0.418
BMI	0.99 (0.94–1.05)	0.805	0.98 (0.92–1.04)	0.486
LAD	1.06 (1.02–1.10)	0.004	1.05 (1.01–1.10)	0.016
Duration of AF persistence	1.05 (0.99–1.10)	0.055	1.03 (0.98–1.08)	0.204
Ablation strategies	1.01 (0.79–1.29)	0.941	0.98 (0.76–1.26)	0.880
FPI in left PVs	0.59 (0.39–0.89)	0.011		
FPI in right PVs	0.58 (0.39–0.87)	0.008		
BOTH	0.62 (0.42–0.92)	0.016		
EITHER	1.04 (0.67–1.61)	0.878		
IFPI	0.47 (0.29–0.74)	0.001	0.46 (0.29–0.74)	0.001

Abbreviations: BMI, body mass index; LAD, left atrium diameter; AF, atrial fibrillation; FPI, first-pass isolation; PV, pulmonary vein; IFPI, at least ipsilateral first-pass isolation; HR, hazard ratio; CI, confident interval.

**Figure 3:**
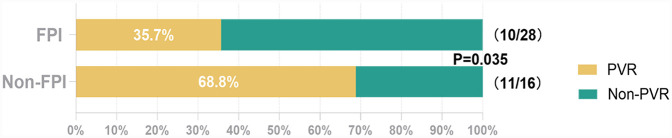
Pulmonary vein reconnection rates in the redo procedure per ipsilateral PVs. Abbreviations: FPI, first-pass isolation; PVR, pulmonary vein reconnection.

Overall, 22 (22.0%) of 100 patients with arrhythmia relapse underwent repeat ablation. The time taken from arrhythmia recurrence to redo procedure was a median of 453 (204, 698) days. Notably, 21 (47.7%) of 44 ipsilateral PVs showed electrical reconnection. The most common site of reconnection was the intervenous carina (56.8%), followed by the posteroinferior aspect (13.6%) and the roof (9.1%) of the circle. The PV reconnection rate in the second procedure was significantly lower in PVs with successful FPI in the first procedure than in others (35.7% vs. 68.8%, *p* = 0.035; Figure 3).

### 3.4 Subgroup analysis of primary endpoint

**Figure 4:**
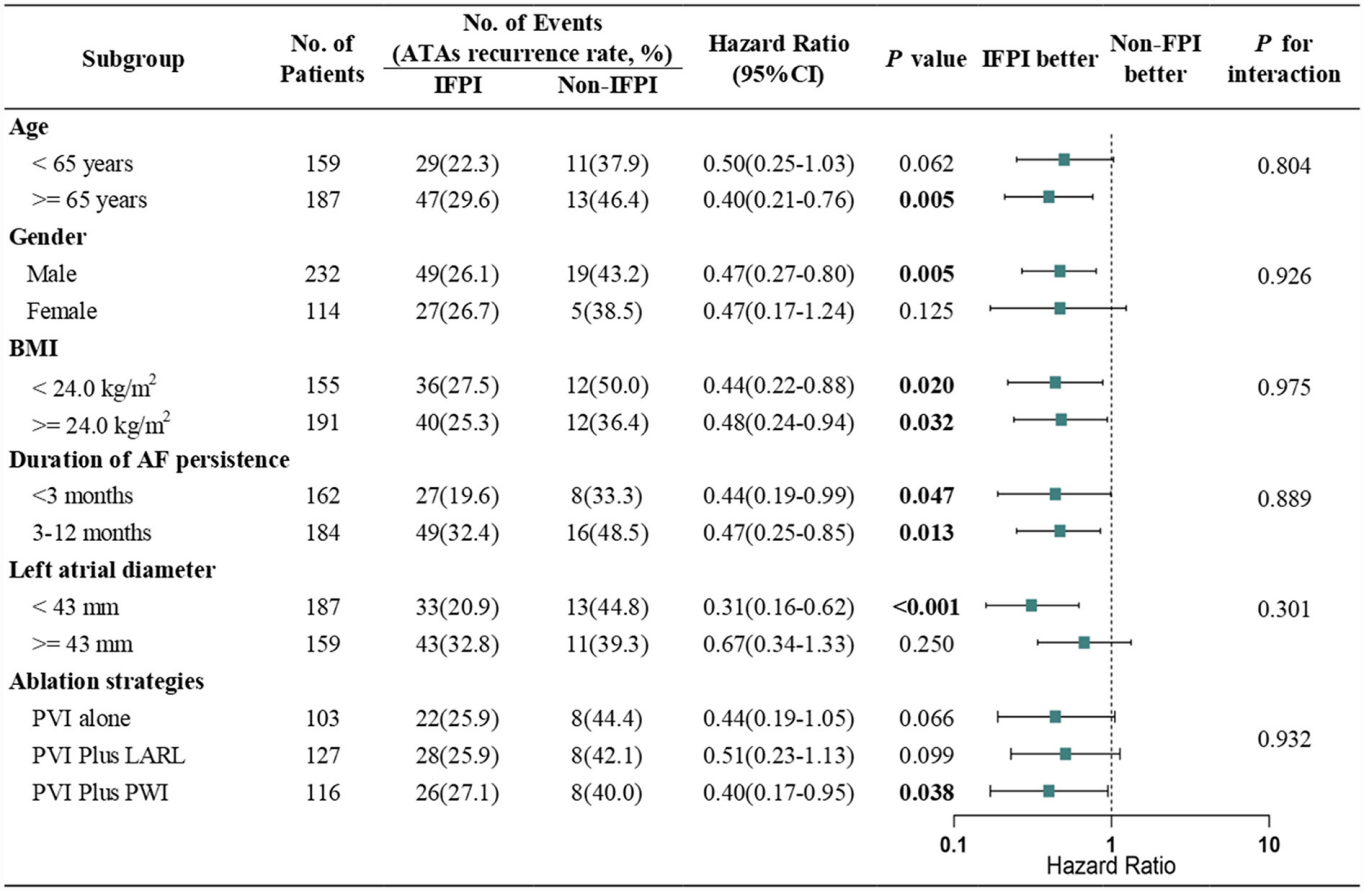
Subgroup analysis of primary endpoint. Hazard ratios and *P* for interaction are based on Cox regression analyses. Abbreviations: BMI, body mass index; AF, atrial fibrillation; PVI, pulmonary vein isolation; LARL, left atrial roof line; PWI, posterior wall isolation.

The risk of ATAs recurrence during the follow­up was evaluated across a range of subgroups, including age, gender, BMI, duration of AF persistence, LAD and different ablation strategies, as depicted in Figure 4. The findings revealed a consistency across all prespecified subgroups, reinforcing the role of IFPI in reducing ATAs recurrence over the non-FPI group.

## 4 Discussion

### 4.1 Main findings

This study provides significant implications for the clinical management of PeAF through the lens of PVI and the achievement of FPI. Our findings suggested several insights: (1) after AI-guided ipsilateral encircling, the site most frequently subjected to touch-up ablation appeared to be the intervenous carina; (2) achieving IFPI was an independent predictor of freedom from ATAs recurrence in PeAF patients undergoing their initial RFA; (3) the prognostic benefit from FPI was primarily applicable to the freedom from recurrence of AF, rather than AT; (4) PV reconnection rate substantially decreased in the second procedure in ipsilateral PVs where FPI was achieved during the initial ablation.

### 4.2 Pulmonary vein isolation as a key success factor

Our study adds to the ongoing discourse surrounding the optimal approach for RFA in PeAF by underscoring the critical role of FPI during PVI. Our findings corroborate the prevailing view that PVI continues to be a cornerstone of successful RFA for PeAF (1). Achieving FPI proved to be a significant factor in enhancing ablation outcomes, as evidenced by the improved freedom from ATAs in our patient cohort successfully undergoing FPI. Contrary to expectations that adjunctive ablation strategies targeting non-PV triggers and substrates could yield better outcomes, our data reflect a lack of compelling evidence supporting these interventions. Despite extensive research over the past two decades, as demonstrated in several renowned randomized controlled trials, there has been negligible impact on reducing recurrent AF rates with additional ablation beyond PVI (3–7). This paradox can be largely attributed to two primary factors: the ambiguity in identifying effective targets for ablation outside the PVs and the technical challenges associated with achieving durable, transmural lesions encircling the PVs. The advent of advanced technologies, such as irrigated RF catheters with contact force-sensing, AI algorithms, and the implementation of the CLOSE protocol, has markedly enhanced the durability and efficacy of PVI. These innovations have facilitated a significant increase in arrhythmia-free survival rates, noted to have risen from approximately 40%–70% at 1-year follow-up in PeAF populations, employing PVI alone (6, 11–14). This substantial improvement highlights the crucial importance of achieving a high-quality lesion set at initial encirclement, as denoted by FPI, thus questioning the necessity of more complex adjunctive strategies. Our study supports the notion that durable PVI is paramount, positing that uncomplicated PVI may suffice in achieving favorable outcomes without the need for complex adjunctive ablation strategies which have not demonstrated clear added benefit in PeAF settings, particularly early-stage PeAF with a normal left atrial substrate.

### 4.3 Impact of first-pass isolation on ablation outcome

FPI has been shown to be associated with a diminished likelihood of acute and chronic pulmonary vein reconnection and favorable clinical results (8, 9, 15, 16). The pathophysiological basis can be attributed to the higher likelihood of creating transmural and contiguous lesions during the initial RF encirclement, thereby reducing the risk of PV reconnection and subsequent AF recurrence. Interestingly, our data reveal that patients from the NEITHER group, who did not achieve FPI in bilateral PVs, exhibited a markedly lower rate of freedom from ATAs. This suggests that the complexity of the left atrial and PV electroanatomy in these patients might impede effective lesion formation, as hypothesized in previous studies (9, 15). The presence of residual conduction gaps and non-transmural lesions, particularly in areas of increased PV wall thickness due to tissue edema, seems to play a crucial role in compromised ablation outcomes (17). Although additional RF applications may sometimes block these epicardial gaps, the transient nature of electrical blocks caused by edema often results in PV reconnection and subsequent AF recurrence (18, 19).

Moreover, the correlation between the absence of FPI and increased AF recurrence, contrasted with a lack of association with AT recurrence, presents a nuanced understanding of AF pathophysiology. Focal triggers originating from PVs are known to be predominant in the early stages of PeAF, especially with a relatively healthier atrial substrate. Consequently, situations where FPI is not achieved allow for easier electrical reconnection between the PVs and the left atrium (8, 15, 16), thereby fostering AF recurrence. In contrast, the recurrence of AT appears to depend less on these initial focal triggers and more on the structural and electrophysiological substrate set by previous ablation lesions (20), indicating a different pathophysiological mechanism. Notably, the study did not find a significant difference in AT recurrence among the different groups, suggesting that FPI's influence may not extend to perturbations in AT etiology. This points to the complex interplay between lesion sets, substrate properties, and arrhythmogenic foci, necessitating further investigation into the mechanistic pathways that could differentiate outcomes for AT and AF post-ablation.

The absence of a statistically significant difference in ablation outcomes between the BOTH group and the EITHER group may stem from multiple interrelated mechanisms: (1) the dominance of unilateral PV triggers in persistent AF, particularly from the left PVs (21–23), may diminish the incremental benefit of bilateral FPI. Anatomically, the left PVs are closer to arrhythmogenic substrates such as the left atrial posterior wall. It can be hypothesized that left PV reconnection is a stronger predictor of AF recurrence than right PV reconnection, but this has not been well documented. In our cohort, left PV FPI success rates were higher than right PVs (72.5% vs. 67.9%), implying that isolating the left PVs alone may suffice to suppress dominant triggers in a subset of patients; (2) Progressive atrial substrate remodeling in PeAF shifts arrhythmia maintenance from PV-dependent triggers to self-sustaining mechanisms (2). Even with bilateral FPI, residual substrate abnormalities could perpetuate AF/AT, reducing the relative advantage of complete PV isolation. This hypothesis is supported by the lack of difference in AT-free survival across groups (Figure 2C), as AT recurrence is more dependent on substrate than PV triggers (20). However, the precise mechanisms underlying this consistency remain elusive and warrant further mechanistic investigation.

It observed that most of touch-up ablation sites in non-FPI PVs were localized to the intervenous carina (Supplementary Figure S1), a region anatomically predisposed to epicardial muscle bundles bridging the LA and PVs (24). The clustering of residual conduction gaps at this epicardial hotspot suggests that incomplete lesion transmurality in this region may underlie FPI failure. Epicardial muscle bundles, which are not fully targeted by endocardial ablation, could perpetuate electrical reconnection and AF recurrence (25, 26). This hypothesis is supported by the significantly higher PV reconnection rate in non-FPI patients (68.8% vs. 35.7%, *P* = 0.035; Figure 3), consistent with prior studies implicating epicardial connections in AF recurrence due to their resistance to endocardial ablation (27). Future prospective studies integrating high-density mapping are needed to directly quantify the role of these pathways in FPI failure.

### 4.4 Perpetuation of atrial fibrillation and its impact on first-pass isolation

The distinct challenges presented by the long-standing nature of AF, especially in cases advancing towards LSPAF, require a nuanced strategy beyond PVI alone (28). As PeAF progresses, the atrial substrate undergoes significant electrical and structural remodeling (2), which can diminish the effectiveness of FPI by extending the pathological mechanisms beyond simple PV triggers. This progression complicates intervention strategies, necessitating the potential incorporation of more aggressive ablation approaches targeting non-PV areas (29, 30). We performed additional statistical analyses for excluded population with LSPAF and found that comparisons amongst the groups (BOTH vs. EITHER vs. NEITHER) revealed no statistically significant difference in the freedom from ATAs recurrence (*P* > 0.05), as expected. This suggests that while FPI is critical, its effectiveness might be compromised in advanced PeAF cases. The findings imply that for advanced cases of PeAF, particularly those approaching LSPAF, optimization of adjunctive ablation strategies might be necessary, even though current evidence regarding these strategies presents a limited prognostic benefit (3–7). Thus, our work not only reinforces the value of achieving FPI but also advocates for targeted exploration into personalized ablation plans as PeAF evolves toward chronicity.

Prolonged duration of AF leads to progressive deterioration of the atrial substrate (31, 32). While our study did not directly assess LA voltage or low-voltage areas (LVA), we propose that LAD, a surrogate for structural remodeling, may reflect underlying substrate heterogeneity. LA enlargement is strongly associated with fibrotic remodeling and larger LVA (33). In our cohort, baseline LAD was comparable across groups (BOTH: 42.5 ± 5.1 mm, EITHER: 42.0 ± 5.7 mm, NEITHER: 42.6 ± 4.3 mm; *P* = 0.666; Table 1), suggesting that differences in LVA burden among groups were likely minimal. However, the relationship between FPI success and LA substrate remains incompletely understood. For instance, Pérez-Pinzón et al. reported that higher LA voltage (indicative of healthier substrate) paradoxically predicted lower FPI rates in the right pulmonary veins, while left PV FPI remained unaffected (34)—a finding that underscores the complex regional interplay between substrate properties and ablation efficacy. Although LVA is a well-established predictor of poor ablation outcomes (35, 36), it remains unclear whether adverse LA substrate directly attenuates the prognostic benefit of FPI or acts as a confounding factor. Larger studies integrating voltage mapping and advanced imaging are needed to dissect these mechanisms.

### 4.5 Study limitation

Several limitations require emphasis in our study. Firstly, this study was retrospective and non-randomized, rendering our conclusions as hypothesis-generating, and selection bias concerning the study population remains a potential concern. Secondly, this study did not explore the impact of FPI on ablation outcomes in patients undergoing additional extra-PV ablation strategies, such as mitral isthmus line, cavotricuspid isthmus line or CFAE ablation, which suggests caution when extrapolating these results to the populations with other ablation strategies. Thirdly, the absence of specific data on the incidence of epicardial connections and LA voltage/fibrosis limited our ability to assess substrate-specific predictors of FPI success; future studies incorporating delayed-enhancement MRI or high-density voltage mapping are warranted to clarify the role of epicardial pathways and substrate characteristics in FPI failure. Fourthly, the lack of continuous cardiac monitoring might have led to an underestimation of arrhythmia recurrence.

## 5 Conclusion

Achieving FPI for PVI is strongly associated with enhanced ablation outcomes in patients with PeAF. The presence of IFPI emerges as a critical predictor for the long-term freedom from ATAs in this patient population undergoing radiofrequency ablation. These findings again highlight the pivotal role of durable PVI in determining successful ablation results for PeAF.

## Data Availability

The raw data supporting the conclusions of this article will be made available by the authors, without undue reservation.
