# Supplementary material for: Impact of the first-pass pulmonary vein isolation on ablation outcomes in persistent atrial fibrillation
Source: Front Cardiovasc Med. 2025 Jun 5;12:1588716. doi: 10.3389/fcvm.2025.1588716 (PMC12176883; doi:10.3389/fcvm.2025.1588716)

Figure S1. Sites of touch-up radiofrequency applications. Touch-up applications in instances of non-first-pass isolation, as depicted from a posteroanterior perspective. Numbers in Fig. indicate the number of patients requiring further radiofrequency applications at these sites.

Abbreviations: LPV, left pulmonary vein; RPV, right pulmonary vein.

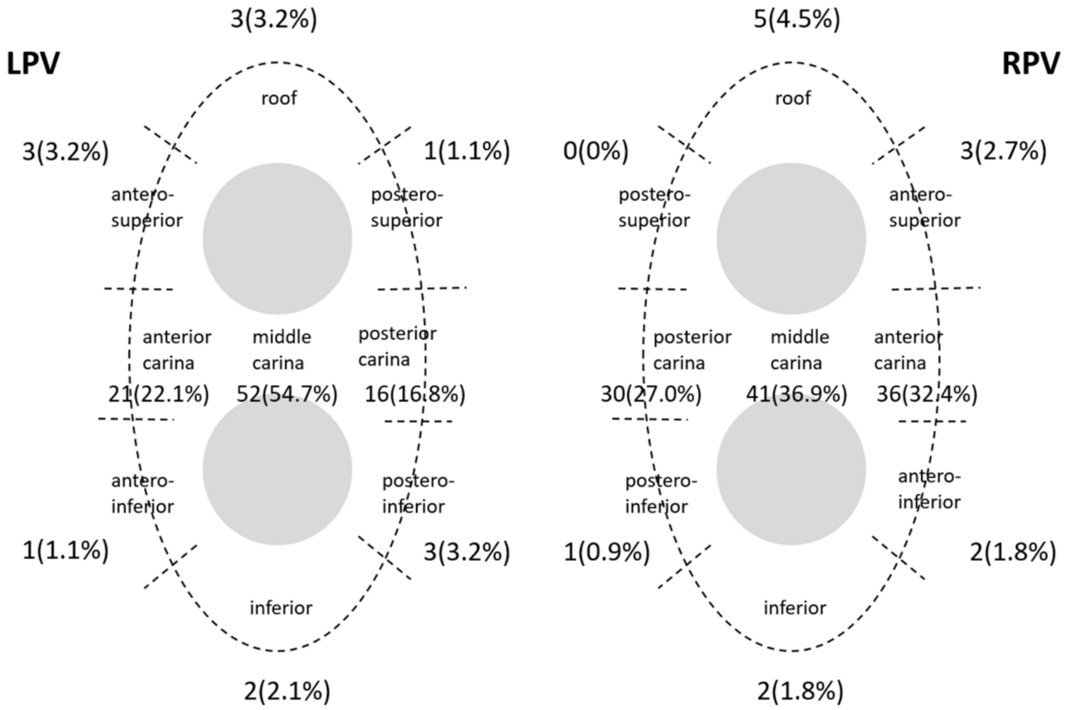

Figure S2. Kaplan-Meier Analysis. Propensity score matching was used to balance baseline characteristics between cohorts. (A) Comparison of ATAs recurrence-free survival between patients treated with PVI alone and those receiving PVI Plus LARL. (B) Comparison of ATAs recurrence-free survival between patients treated with PVI alone and those receiving PVI Plus PWI.

Abbreviations: ATAs, atrial tachyarrhythmias; PVI, pulmonary vein isolation; LARL, left atrial roof line; PWI, posterior wall isolation.

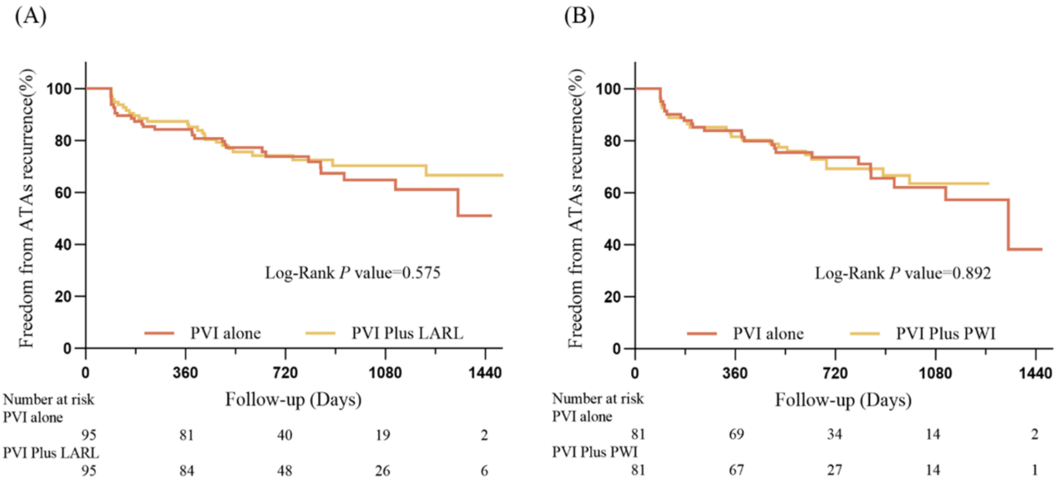

Supplement: Supplementary file 1 [file Datasheet1.pdf]
